# MAF-MixNet: Few-Shot Tea Disease Detection Based on Mixed Attention and Multi-Path Feature Fusion

**DOI:** 10.3390/plants14081259

**Published:** 2025-04-21

**Authors:** Wenjing Zhang, Ke Tan, Han Wang, Di Hu, Haibo Pu

**Affiliations:** College of Information Engineering, Sichuan Agricultural University, Ya’an 625014, China; 202205963@stu.sicau.edu.cn (W.Z.); tanke@stu.sicau.edu.cn (K.T.); 202205605@stu.sicau.edu.cn (H.W.); hudi@stu.sicau.edu.cn (D.H.)

**Keywords:** tea disease detection, attention mechanism, feature fusion, few-shot object detection

## Abstract

Tea (*Camellia sinensis* L.) disease detection in complex field conditions faces significant challenges due to the scarcity of labeled data. While current mainstream visual deep learning algorithms depend on large-scale curated datasets. To address this, we propose a novel few-shot end-to-end detection network called MAF-MixNet that achieves robust detection with minimal annotation data. The network effectively overcomes the bottleneck of insufficient feature extraction under limited samples of existing methods, through the design of a mixed attention branch (MA-Branch) and a multi-path feature fusion module (MAFM). The former extracts contextual features, while the latter combines and enhances the local and global features. The entire model uses a two-stage paradigm to pretrain on public datasets and fine-tune on balanced subset datasets, including novel tea disease classes, anthracnose, and brown blight. Comparative experiments with six models on four evaluation metrics verified the advancement of our model. At 5-shot, MAF-MixNet achieves scores of 62.0%, 60.1%, and 65.9% in precision, nAP50, and F1 score, respectively, significantly outperforming other models. Similar superiority is achieved in the 10-shot scenario, where nAP50 is 73.8%. Our model maintains a certain computational efficiency and achieves the second fastest inference speed at 11.63 FPS, making it viable for real-world deployment. The results confirm MAF-MixNet’s potential to enable cost-effective, intelligent disease monitoring in precision agriculture.

## 1. Introduction

*Tea* (*Camellia sinensis* L.) is the second most consumed beverage in the world after water. The production and processing of *tea* not only strongly boost social and economic development but also promote global trade. However, some typical tea diseases can greatly reduce *tea*’s antioxidant activity, healthcare function, and commercial value. Failure to control and handle the spread of diseases in a timely and effective manner will also lead to reduced production and income [1]. Traditional tea disease detection mainly relies on manual inspection, and this labor-intensive method shows low accuracy due to visual fatigue and subjectivity of discrimination [2]. With the continuous development and application of artificial intelligence technology, especially deep learning technology, tea disease detection has gradually shown an intelligent and de-artificialization development trend [3,4,5].

Most of the current tea disease detection methods use common object detection or semantic segmentation methods that require a large amount of labeled data. Md Janibul Alam Soeb et al. [5] implemented high-performance detection of five types of diseases in natural environments based on a single-branch You Only Look Once (YOLO) network, but the amount of labeled data required reached 4000 pieces. Ji Li and Chenyi Liao [6] proposed a two-stage semantic segmentation network combined with a generative adversarial network to deal with disease detection in complex weather and lighting environments. Gensheng Hu et al. [7] proposed an elliptical recovery method to simulate occlusion and damaged leaves, combined with the U-Net network and Support Vector Machine (SVM) classifier to evaluate the severity of tea blight. Although the number of disease data per class in the datasets they used is below 100, considering the complexity of data annotation for semantic segmentation tasks, a lot of time is also needed for preliminary data processing. From a macro perspective, these methods do not reduce the time and effort spent on disease detection but only shift the allocation of such resources to different stages. Few-shot learning has been widely used as a solution to effectively alleviate the dependence on large-scale labeled data and realize the rapid generalization of disease characteristics under limited labeled samples. Few-shot learning aims to imitate the ability of humans to quickly summarize and learn from a small number of examples. In the field of crop disease detection, Masoud Rezaei et al. [8] adopted a meta-learning method to alleviate the problem of data scarcity. Through pre-training, meta-learning, and fine-tuning, an accuracy of over 90% was achieved in the classification task of 5 images per class of 5 types of diseases. In this work, the Vision Transformer backbone network and the feature attention mechanism in the prototype network are mainly used to obtain features, but the fusion of global and local information is ignored. In the object detection task, more coordination of the two types of information is needed to assist in positioning. Therefore, this method may have the problem of decreased accuracy in the object detection task. Yang Li and Xuewei Chao [9] proposed the first method of setting pseudo-labels for semi-supervised learning on a crop disease dataset. However, this method only uses a pure convolutional structure, and the extracted features are not rich enough. In the field of tea disease detection, Xianze Yao et al. [10] used a transfer learning combined with data augmentation to achieve few-shot detection and solved the problem of small targets by decoupling the detection head and introducing a Triplet Attention. However, this method ignores the extraction of long-range dependencies. Although multi-scale features are extracted by the final decoupled detection head, there is a lack of interaction and fusion between the features. Gensheng Hu et al. [11] proposed a method that first segments the disease area, then uses C-DCGAN to generate and expand the training samples, and finally locates the disease, reaching an average accuracy rate of 90% on a dataset of 3 types of diseases with 20 samples per class. Although the generative model increases the diversity of data, the generated images no longer have complex backgrounds after segmentation, and the training model may not be suitable for real-world production. The detection network itself does not extract disease spot features well enough, failing to focus on the low-level detailed information and the overall shape of the leaves. This method involves the training of three sub-networks, making the training process more difficult and consuming more computation.

To address the above issues, this paper proposes a few-shot end-to-end MAF-MixNet network for detecting tea anthracnose and brown blight. The model integrates local and global feature extraction with critical feature enhancement. The innovations of the proposed method are reflected in three aspects:

1. A mixed attention branch (MA-Branch) is proposed to extract global semantic information, which works in parallel with traditional convolutional layers. It realizes context awareness by coordinating a multi-head self-attention mechanism, a spatial attention mechanism, and a channel attention mechanism;
2. A multi-path fusion module (MAFM) is proposed to calibrate and jointly enhance the feature representations of dual paths in a nonlinear adaptive manner. This significantly improves model performance while introducing minimal additional computational parameters;
3. We have created a real scene leaf disease detection dataset dedicated to few-shot learning by employing data cleaning and data augmentation strategies. The data augmentation is used for improving the data size and the model’s generalization ability through simulating natural lighting conditions. The dataset supports progressive dataset partitioning and enriches the existing few-shot dataset system based on base class and new class training paradigms.

This paper provides a novel and effective method for detecting tea diseases, providing technical support for the field of precision agriculture. In addition, this study can also provide a useful reference for the detection of other crop diseases and plays a positive role in promoting the application of artificial intelligence technology in agricultural production.

## 2. Related Work

### 2.1. Attention Mechanism

The attention mechanism is a method that makes the model focus on important areas of the image while ignoring irrelevant areas. This mechanism is achieved by assigning different importance weights to different parts of the input, such as channels, space, time sequence, and other dimensions [12]. For a given input *X*, we can simply represent the attention mechanism as

(1) $$\begin{array}{c} \mathrm{Attention}=f(g(X),X) \end{array}$$

g(·) represents the operation of computing the attention weights for *X*, where different attention mechanisms employ distinct methods to derive these weights. This operation finds the similarity between the query and the key for the self-attention mechanism and transforms it into a probability distribution. f(·) corresponds to the process of getting key information from the input. The attention mechanism has been applied in many fields because of its efficient feature extraction ability. Yunjie Tian et al. [13] propose a novel area attention module to extract global features in a large receptive field effectively. It eliminates the shortcomings of linear attention mechanisms and local attention mechanisms, requiring only a reshaped operation to achieve faster speed and lower computational cost. Among them, the Transformer module, which takes the self-attention mechanism as a key part, shows not only outstanding results in the field of Natural Language Processing [14,15] but also makes outstanding contributions in the field of Computer Vision [16,17]. Currently, Zhixuan Lin et al. [18] add the forget gate into transformers to further improve their ability to filter essential information. Debasish Dutta et al. [19] show that the transformer-based methods have surpassed Convolutional Neural Network (CNN) and GAN-based methods in the image super-resolution task. Additionally, Yuqi Yang et al. [20] design a 3D Swin Transformer backbone, enabling efficient self-attention on sparse voxels. The Transformer has become a new structure that is expected to replace convolution. In the field of few-shot object detection, by introducing an attention mechanism, the model can make full use of a small amount of sample information to make more accurate classification and positioning. Guangxing Han et al. [21] introduced multi-layer asymmetric batch cross-attention to two-branch networks, aggregating query features and supporting features and improving the effect of feature alignment. Qi Fan et al. [22] added an attention mechanism to the region proposal network (RPN) to avoid generating a large number of candidate proposals that do not contain potential targets as much as possible so that the network can focus more on foreground information. Masoud Rezaei et al. [23] used a Swin Transformer as the network backbone to apply few-shot disease detection on barley plants. Sai Babu Veesam et al. [24] proposed a transformer-augmented Recurrent Neural Network (RNN) to improve the original RNNs’ temporal modeling ability and apply the prototypical networks to generalize to a few examples. Wei Liu et al. [25] designed a novel mutual attention feature enhancement module to diagnose anomalies in high-speed trains under few-shot learning. In the task of tea disease detection in complex backgrounds, we use a variety of attention mechanisms to focus on disease areas and improve disease detection capabilities on the basis of single-branch feature extraction with pure convolution.

### 2.2. Feature Fusion

Feature fusion is a technique to combine the outputs of different branches or layers. Feature fusion is mainly used to enhance feature representation, especially when dealing with multi-scale features, cross-modal tasks, and the need to fuse information from multiple sources. Simple feature fusion methods usually use linear aggregation methods, such as addition or concatenation. This fixed aggregation method may not be suitable for all feature objects. With the rise of attention mechanisms, feature fusion methods have gradually become dynamic. According to the feature map, adaptive fusion and continuous iterative correction [26]. By fusing features in the potential space, Xiaowei Yu et al. [27] improve the robustness and generalization ability of the model to noise. Jin Ding and Xue Zhou [28] proposed a learnable feature fusion module that fuses global and local features based on a teacher-student training strategy, avoiding the ambiguity problem in multi-pseudo-label learning. However, this method is limited by the fact that it does not perform deep extraction of global features, which may introduce noise information in complex backgrounds and affect the performance of the model. José Morano et al. [29] proposed the first layer-level feature fusion framework and two novel fusion approaches for 3D to 2D. However, the quality of the fusion largely relies on the quality of the medical images. Hui Li and Xiaojun Wu [30] proposed a hybrid network architecture combining CNN and Transformer to enhance complementary information between different modalities. It achieves state-of-the-art performance in infrared-visible image fusion tasks. In the field of few-shot learning, feature fusion can alleviate data insufficiency and suppress model overfitting. Guangxing Han et al. [31] designed a lightweight nonlinear feature fusion network (combining multiplication, subtraction, and concatenation operations) to dynamically fuse multi-source features in the proposals generation (Meta-RPN) and classification (Meta-Classifier) stages, addressing the issues of inaccurate feature similarity calculation and spatial misalignment in few-shot object detection and significantly improving detection performance. The shortcoming of this approach is that the nonlinear fusion method lacks adaptability to changes in the distribution of different features. Songtao Liu et al. [32] proposed an adaptive spatial fusion module for pyramid features, which improves the scale invariance of features by learning spatial filtering to suppress the consistency problem between different feature scales. However, in this method, the feature maps of different layers are fused by directly adjusting the size, which may lose some feature information. Israr Hussain et al. [33] proposed the first few-shot approach for recaptured image detection. They design three different parallel feature fusion modules to improve feature representation and highlight significant features. In this paper, we use the adaptive feature fusion method to enhance the expression of global and local key information and improve detection accuracy in few-shot scenarios.

### 2.3. Few-Shot Object Detection

Few-shot object detection (FSOD) aims to achieve the classification and localization of objects in images with a small number of labeled samples. In terms of form, the training data of few-shot object detection can be divided into the base class dataset $D_{base}$ and the novel class dataset $D_{novel}$. The base class contains a large amount of labeled information, while the new class has very little labeled data, and there is no intersection of data types between the two datasets. To avoid overfitting and improve the generalization ability of the model as much as possible, current few-shot object detection methods usually train the base class detection model $M_{base}$ on the base class dataset and then fine-tune it using the novel class dataset to obtain the final detection model $M_{final}$. Common training methods include transfer learning, metric learning, and meta-learning. Transfer learning refers to the use of model weights that have been trained on a large dataset before detecting novel classes. Since the model’s detection performance is related to the model’s initial weights to some extent, the transfer learning method is equivalent to giving the model a good start and improving the detection efficiency. Metric learning is learning a data representation so that similar inputs have a closer metric distance, and conversely, the farther apart the metric distance. Meta-learning is a method that allows models to learn to learn. After the model learns knowledge related to the target domain on a large-scale data set, it can efficiently learn detection knowledge on a few sample data [34].

(2) $$\begin{array}{c} M_{init}\overset{D_{base}}{\to }M_{base}\overset{D_{finetune}}{\to }M_{final} \end{array}$$

Based on the model structure, few-shot object detection methods can be divided into single-branch methods [35,36,37,38] and two-branch methods [21,22,39]. The single-branch method is similar to the structure of the general detector but can reduce the number of learnable parameters when training for new categories. Bo Sun et al. [35] introduced a contrastive head at the end of the network to measure the similarity of object proposals, reduce the variance between proposals of the same class of targets, and improve the accuracy of classification. The two-branch method is based on the Siamese network for query and support. Guangxing Han et al. [39] promoted the adaptation between the two branches through the heterogeneous graph convolutional neural network. In this paper, based on the single-branch structure network, we use the transfer learning method with a pre-training and fine-tuning paradigm to solve the task of tea disease detection and realize accurate disease detection with few samples.

**Figure 1:**
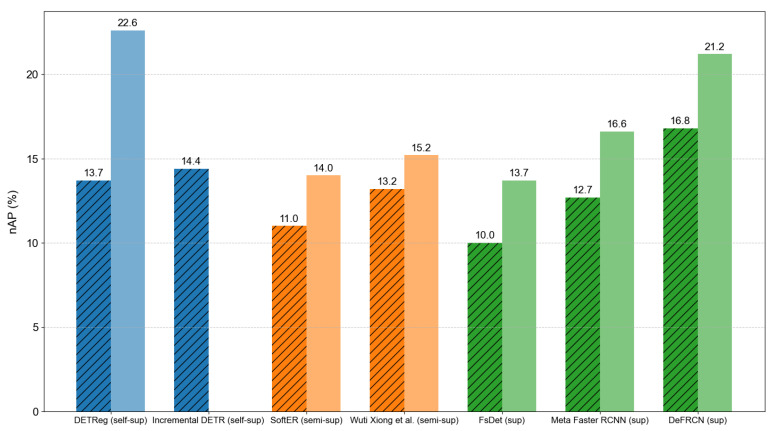
Comparison of pre-training methods in few-shot object detection. The blue, orange, and green columns represent self-supervised, semi-supervised, and supervised methods, respectively. Dark and diagonal represent 10-shot scenarios, and light colors represent 30-shot scenarios. All data are from the original literature.

Recently, a variety of research methods have emerged in the field of few-shot object detection, which combines traditional few-shot learning methods with novel methods in other fields. It is worth mentioning that many current methods have improved the bottleneck of the pre-training stage in the pre-training and fine-tuning paradigm and have tried self-supervised [40,41,42] and semi-supervised [43,44] methods to further alleviate the dependence on labeled data. Because the labels may be limited in both base classes and novel classes in real scenarios, many researchers are exploring other methods to advance few-shot object detection. Self-supervised learning is one of the alternatives. Usually, pretext tasks are used in the pre-training stage to train large-scale unlabeled data, thus making the model learn a representation. Then, migrate to downstream tasks in the fine-tuning stage. Amir Bar et al. [41] introduced a self-supervised method that pre-trains the whole detection network using an unsupervised region proposal generator and a self-supervised image encoder. And then perform fine-tuning in the few-shot setting. Semi-supervised learning lies between supervised learning and unsupervised learning. The current state-of-the-art in semi-supervised learning mainly uses the pseudo-labeling method. It’s a method that trains a model on a labeled dataset and then uses it to predict the labels for the unlabeled dataset. Phi Vu Tran [44] proposed a robust detector that combines pseudo-labeling and consistency learning on region proposals to tackle the scenario that both base and novel labels are scarce. We further draw a plot to compare these three methods, as shown in Figure 1. The semi-supervised and self-supervised approaches are mainly used in image classification tasks, and there are few works that focus on instance-level tasks, like object detection and semantic segmentation [40]. Although the two alternative methods reduce the dependence of the base classes on labeled data, they do not have a significant advantage over the supervised method in terms of performance. This indicates that there is still a long way to go. In our work, we still adopt supervised pre-training; however, we plan to explore self-supervised or semi-supervised methods in future iterations.

## 3. Materials and Methods

### 3.1. Dataset Collection

The plant leaf disease detection data set constructed in this study is partly self-collected data and partly from the network. Among them, tea disease data were collected from a tea garden in Ya’an City, Sichuan Province, China, covering two typical leaf diseases: tea anthracnose disease and tea brown blight disease. The other part of the data includes cotton fusarium wilt disease (*Fusarium oxysporum* Schl.sp.vasinfectum (Atk.) Snyder et Hansen) and cotton powdery mildew (*Leveillula malvacearum* Golov.). The above diseases are common diseases in agricultural planting and production, which are highly contagious and easy to lead to yield reduction and quality deterioration. Traditional chemical control methods are at risk of environmental pollution and limited control effects, while artificial control strategies will require significant amounts of energy and financial resources. Therefore, precise and non-destructive detection based on computer vision provides a sustainable and convenient solution that can help build a precise agricultural management system.

**Figure 2:**
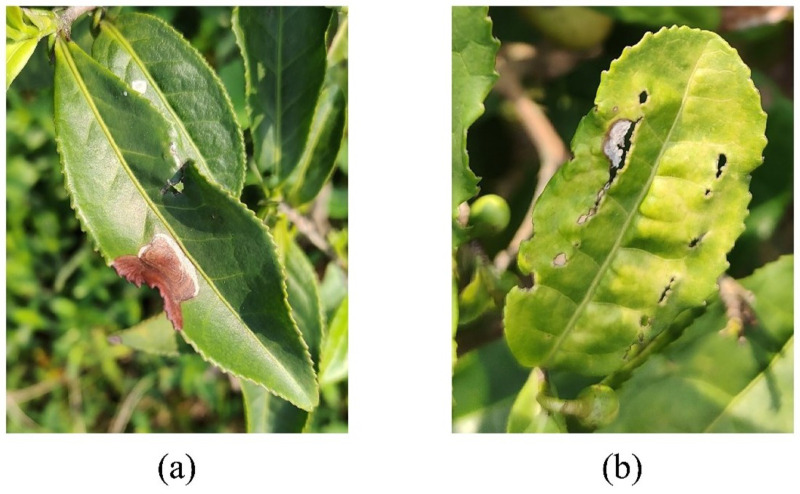
Data samples of tea diseases. (**a**) Tea brown blight, (**b**) Tea anthracnose.

**Figure 3:**
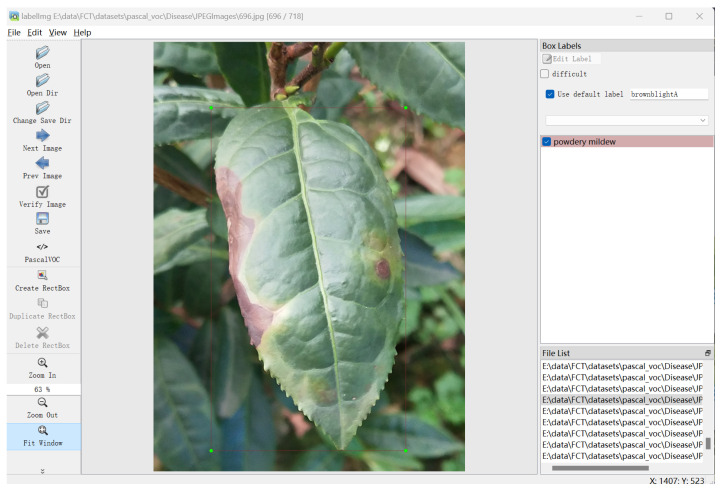
Illustration of the process of data labeling.

To ensure the applicability of the model in the actual agricultural production environment, the data in the dataset are all real scene images with diseased leaves as the main body and a complex field background. The collection of tea disease data was carried out at the end of spring and the beginning of summer, which is the incidence period of tea diseases. The image acquisition equipment has a macro camera with 2 million pixels. In order to ensure the clarity of the image, the image acquisition work was carried out on sunny days with sufficient light. The distance between the camera and the leaves is about 5–10 cm to ensure the complete presentation of the leaf, a clear leaf outline, and an obvious disease infection area. Figure 2 presents the *tea* data samples and Figure 3 is the annotation process. It is worth noting that in actual agricultural production, farmers often take immediate action to remove when they find diseased leaves, resulting in the scarcity of tea disease samples. To this end, this study innovatively constructs a hybrid data strategy. On the basis of independently collecting high-quality field samples, open-source disease image resources on the Internet are integrated. This method has two advantages: Firstly, it improves the richness and diversity of the data. Secondly, using the fine-tuning strategy in transfer learning, the model can be pre-trained on a large-scale online leaf disease dataset to learn general features and then realize few-shot tea disease detection through subsequent fine-tuning. Therefore, based on cotton disease categories as the basic class $C_{b}$, tea disease types are novel classes $C_{n}$. The distribution of the original dataset is shown in Table 1.

### 3.2. Data Preprocessing

Our data preprocessing procedure includes data cleaning and data augmentation. In the process of agricultural image data acquisition, due to equipment limitations, environmental interference, improper operation, and other reasons, there are often low-quality images in the dataset, such as blurred or redundant samples. Such data can easily lead to overfitting, feature confusion, and reduced generalization during model training. Therefore, it is necessary to carry out strict data cleaning to construct a high-quality plant disease detection dataset:

1. **Cleaning of blurred images:** The blurring and distortion of leaf disease images are mainly caused by camera shake, focusing errors, or environmental interference (such as wind or lighting changes) during the acquisition process. The artifacts in such images can convey misleading information and cause the model to learn incorrect features during training. Meanwhile, the lack of high-frequency information significantly reduces the model’s ability to distinguish disease edge features, resulting in poor performance in practical applications.
  (3) $$\begin{array}{c} \nabla^{2}I\left(x,y\right)=\frac{\partial^{2}I}{\partial x^{2}}+\frac{\partial^{2}I}{\partial y^{2}} \end{array}$$
  (4) $$\begin{array}{c} \mathrm{Var}\left(\nabla^{2}I\right)=\frac{1}{N}\sum_{x,y}{\left(\nabla^{2}I\left(x,y\right)-\mu \right)}^{2} \end{array}$$
  To eliminate the ambiguity of the image, we use the Laplacian operator variance method to quantify the clarity of the image. The Laplacian operator can highlight image areas that contain rapid intensity changes by calculating the second derivative of the image. Similar to the Sobel and Scharr operators, it is commonly used for edge detection. A high variance indicates that the image has rich high-frequency details; that is, its edge features and texture details are more significant, corresponding to the frequency domain features of a clear image in a normal focus state. Conversely, a low variance value indicates that the high-frequency information in the image is missing, and its intensity distribution tends to be uniform. Therefore, the lower the Laplacian variance value, the more blurred the image is. Through experiments and analysis, it was found that a threshold of 120 can effectively distinguish clear images from blurred images. The specific calculation formulas are shown in Formulas (3) and (4), where I(x,y) is the gray value of the image at (x,y), μ is the mean value of the Laplacian image, and *N* is the total number of pixels in the image.
2. **Cleaning of redundant images:** Redundant images primarily result from continuous shooting or repeated acquisition, which exhibit high visual similarity and may contain nearly identical disease areas and background information. These redundant images can lead to model overfitting during training and reduce its discriminative capability for disease features. To eliminate redundancy, we employ the Structural Similarity Index (SSIM) to measure inter-image similarity. SSIM evaluates image similarity through three components: luminance similarity, contrast similarity, and structural similarity. Specifically, it uses means to estimate luminance similarity, standard deviations for contrast similarity, and covariance to measure structural similarity. The final similarity score is the product of these three components, ranging from −1 to 1, where an SSIM value of 1 indicates identical images. We set the threshold at 0.85, which is used in many methods. When the structural similarity between two images exceeds 0.85, one of them is randomly deleted. The detailed SSIM calculation formula is shown in the following equation:
  (5) $$\begin{array}{c} \mathrm{SSIM}\left(x,y\right)=\frac{\left(2\mu_{x}\mu_{y}+c_{1}\right)\left(2\sigma_{xy}+c_{2}\right)}{\left(\mu_{x}^{2}+\mu_{y}^{2}+c_{1}\right)\left(\sigma_{x}^{2}+\sigma_{y}^{2}+c_{2}\right)} \end{array}$$
  *x* and *y* represent the two images being compared, $\mu_{x}$ represents the average brightness of image *x*, $\sigma_{x}$ represents the standard deviation of image *x*, and $\sigma_{xy}$ represents the covariance between images *x* and *y*. This formula quantifies the level of structural similarity between the two images.

After applying the above data cleaning steps, the original disease data set contains 496 images. After deleting fuzzy images and redundant images, 201 high-quality images were retained. In order to further improve the quality of the data, it is still necessary to manually select abnormal images and images with classification errors, which is very necessary for the subsequent training of the deep learning model. After manually deleting 48 image data, 153 image data were retained for subsequent data augmentation procedures, as shown in Table 2.

Due to the inevitable reduction in sample size caused by data cleaning, it is not conducive to obtaining a model with strong generalization ability and is not easy to overfit in the pre-training stage. Therefore, data enhancement technology is still needed to carry out a certain degree of data expansion. Common data enhancement methods include geometric transformation and pixel transformation. We first use photometric transformation in pixel transformation to simulate the samples collected under different weather conditions. By changing the contrast and brightness of pixels, the cotton leaf disease data used for pre-training has been expanded. The mathematical expression is as follows:

(6) $$\begin{array}{c} I^{\prime }\left(x,y\right)=clip\left(\alpha \cdot I\left(x,y\right)+\beta ,0,255\right) \end{array}$$

Among the parameters, α controls the image contrast. When α>1, the contrast is enhanced, thus sharpening the details. When it is between 0 and 1, the contrast decreases. β adjusts the brightness offset, and a value greater than 0 makes the image brighter, while a negative value makes it darker. The clip(·) function is used to ensure that the pixel values are within the valid range of 0 to 255. By combining the parameters α=1.2 and β=8, the situation of sufficient sunlight on sunny days is simulated. α=0.8 and β=−10 are set to simulate rainy days. In this way, the original dataset is expanded by two times. On this basis, uniform noise is added to the image to generate new samples. Adding noise can increase the diversity of data, improve the robustness of the model, and prevent the model from overfitting. The enhanced samples can be defined in the following form:

(7) $$\begin{array}{c} I_{noisy}\left(x,y\right)=clip\left(I\left(x,y\right)+\Delta \cdot U\left(-\theta ,\theta \right),0,255\right) \end{array}$$

U(−θ,θ) indicates a uniform distribution in the interval [−θ,θ], where θ=0.25. The symbol Δ represents the pixel value scaling factor, which is fixed at 255. At this point, the enhancement of cotton leaves is achieved, and the enhancement results are shown in Table 3. The final data set has 718 samples, of which 40 are *tea* samples.

### 3.3. Proposed Method

#### 3.3.1. Overall

**Figure 4:**
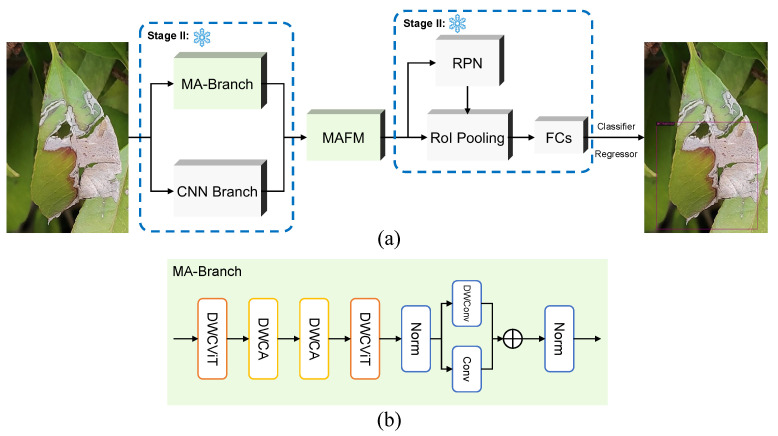
Overview of the proposed method: (**a**) Architecture of the MAF-MixNet model. The right picture of the tea leaf shows the detection result; (**b**) Structure of the Mixed Attention Branch (MA-Branch). In the stage two, we only fine-tune the MAFM and heads, remaining the other modules frozen.

Aiming at the problem of representation limitation of traditional object detection networks in the scenario of scarce tea disease samples, this paper proposes a dual-branch collaborative feature enhancement network, as shown in Figure 4. Its core architecture includes a mixed attention branch (MA-Branch) and a multi-path feature fusion module (MAFM). Although the backbone of a traditional convolutional neural network can use the characteristics of local perception and spatial invariance of convolution to obtain the feature map of the input image for further analysis through a variety of combinations of convolution (such as residual connection, jump connection, etc.), for the current task, the supervised data is scarce, and the full convolutional structure cannot extract sufficient features. To solve this problem, we design a mixed attention branch in parallel based on retaining the traditional convolution branch. In this branch, we integrate a multi-head self-attention mechanisms and content-guided attention mechanisms through **D**epthwise **W**eighted **C**onvolutional **Vi**sion **T**ransformer (DWCViT) and **D**epthwise **W**eighted **C**onvolutional **A**ttention (DWCA) modules to establish long-range semantic correlation across regions and strengthen critical features. Then, we design a multi-path feature fusion module to make full use of the information extracted by the two branches. MAFM adopts an adaptive weighted strategy to realize the deep integration of local details and global context. After deep fusion, the features of the two branches generate enhanced features with both global semantic consistency and local accuracy. Region Proposal Network (RPN) [45] uses enhanced features to generate candidate proposals, which are classified and regressed after RoI Pooling, and finally realize end-to-end object detection under a few-shot setting.

We adopt a two-stage fine-tuning method to train the above few-shot object detection network following previous work in [36]. Based on the disease dataset D=(x,y),x∈X,y∈Y constructed in Section 3.1 and Section 3.2, which *x* refers to the input image, $y=(c_{i},I_{i}),i=1,\ldots ,N$, $c_{i}$ and $I_{i}$ respectively represent the category label and bounding box data of N target instances in the image *x*, $c\in C_{b}⋃C_{n}$ contains the base classes and the novel classes. In the first stage, the base model training stage, we use the base class $C_{b}$ data to train the detector from scratch. The optimization object is a joint loss.

(8) $$\begin{array}{c} L=L_{cls}+L_{rpn}+L_{loc} \end{array}$$

$L_{cls}$ is a cross-entropy loss. $L_{rpn}$ is used to tell the foreground from the background and refine the anchors. $L_{loc}$ is a smooth L1 Loss. By driving the network to complete parameter optimization with rich sample data in the base classes, the model is enabled to establish a general representation ability of plant diseases. In the fine-tuning stage, we construct a small sample balanced data set for training based on the base classes and the novel classes, with K-shot for each category. Thus, the model is transferred to novel classes while avoiding forgetting the base classes. At this time, only the feature fusion module and the detection head are unfrozen so that the model can learn the features related to the target task and make full use of the prior knowledge. The loss function used for classification in this stage is different from the pretraining stage. Following [36], we use a cosine similarity for the box classifier, which is proven to improve the detection accuracy. This method can effectively solve the problem of insufficient representation learning due to data scarcity and improve the accuracy of tea disease detection and the generalization of the model while maintaining the discrimination ability of the base classes.

The proposed method has the following advantages:

**(1) Two-branch feature collaboration:** while retaining the local feature extraction capabilities of convolution, various attention mechanisms, such as self-attention mechanism and the spatial attention mechanism, are introduced to improve the model’s attention to global semantics and highlight significant features.

**(2) Multi-path adaptive feature fusion:** the proposed MAFM realizes the optimal coupling of local detail features and global information. It helps to improve detection performance while introducing a few parameters.

**(3) Few-shot Object Detection Performance Enhancement:** employing a training strategy that combines pre-training with partial parameter fine-tuning can mitigate overfitting risks and synergistically optimize both model generalization capability and computational efficiency.

#### 3.3.2. MA-Branch

The introduction of the mixed attention branch aims to improve the feature extraction ability of the network in the case of few-shot and complex detection backgrounds. It mainly includes two modules: DWCViT and DWCA. The design and working principle of these two modules will be elaborated in the following text.

**Figure 5:**
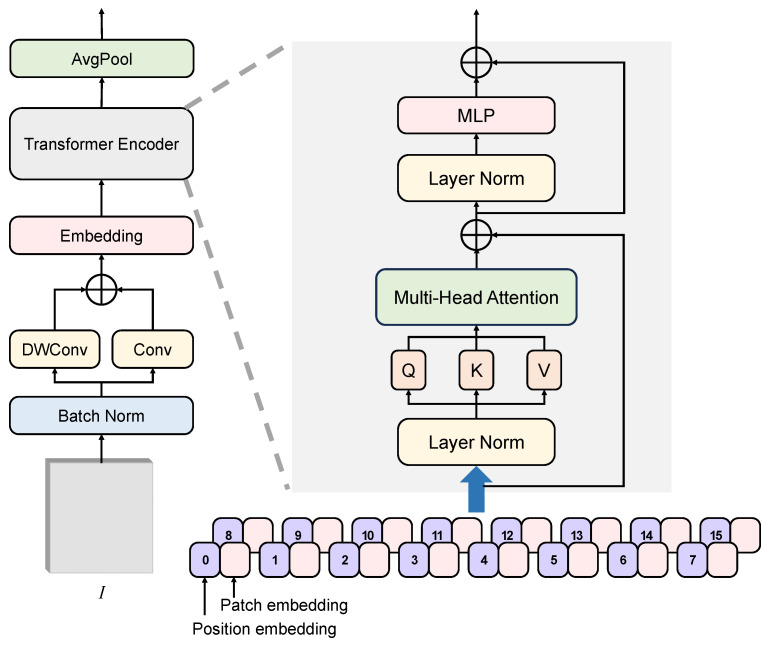
Structure of DWCViT. The ⨁ represents pointwise addition.

Unlike traditional Vision Transformers that directly processes raw images, the DWCViT module couples depth-wise separable convolutions, standard convolutions, and attention mechanisms to mitigate the lack of local inductive bias in Vision Transformers while reducing computational complexity [46,47]. Taking the first DWCViT module as an example for a detailed explanation, its structure is shown in Figure 5. Given an input image $I\in R^{B*H_{I}*W_{I}*3}$ (where *B* is the batch size, $H_{I}$ and $W_{I}$ are the image height and width, respectively), the process begins with channel-wise normalization through a batch normalization layer. Subsequently, image features are extracted via a depth-wise separable convolution and a standard convolution, followed by element-wise summation to obtain the feature $F\;\in \;R^{B*H_{I}*W_{I}*3}$

(9) $$\begin{array}{c} F=DWConv(BN(I))+Conv(BN(I)) \end{array}$$

BN(·) denotes batch normalization. DWConv(·) represents a depth-wise separable convolution, composed of a 3×3 depth-wise convolution followed by a 1×1 pointwise convolution. Subsequently, following the original Vision Transformer architecture, the feature map F is divided into 16×16×3 image patches. These patches are then flattened and projected to produce the patch embedding sequence $X_{f}\in R^{N_{f}*C}$, where $N_{f}=\frac{H_{I}}{16}*\frac{W_{I}}{16}$ (number of patches) and C=16∗16∗3 (embedding dimension per patch). To preserve spatial positional information, a learnable positional encoding $E_{f}^{pos}\in R^{N_{f}*C}$ is introduced and added to the image embeddings. Finally, the position-augmented embedding sequence $X_{f}^{\prime }$ is fed into a transformer encoder composed of a multi-head self-attention mechanism and feed-forward network, enabling global feature extraction and semantic information acquisition. The encoder output sequence $X_{o}\in R^{N_{f}*C}$ is reshaped back into a 2D feature map format $F^{\prime }\;\in \;R^{B*H_{I}*W_{I}*3}$ for downstream modules. The core computation of this process can be formulated as follows:

(10) $$\begin{array}{c} X_{f}^{\prime }=X_{f}+E_{f}^{pos} \end{array}$$

(11) $$\begin{array}{c} Attention\left(Q,K,V\right)=Softmax\left(QK^{T}/\sqrt{d}\right)V \end{array}$$

(12) $$\begin{array}{c} head_{i}=Attention\left(W_{i}^{Q}\cdot X_{f}^{\prime },W_{i}^{K}\cdot X_{f}^{\prime },W_{i}^{V}\cdot X_{f}^{\prime }\right) \end{array}$$

(13) $$\begin{array}{c} MHA\left(Q,K,V\right)=Concat\left(head_{1},\ldots ,head_{h}\right)W^{0} \end{array}$$

In Equation (11) of the attention mechanism, the attention scores are computed by multiplying the query matrix *Q* with the transposed key matrix $K^{T}$. These scores are then scaled by dividing by $\sqrt{d}$ (where $d=\frac{C}{h}$, with *h* being the number of attention heads) to prevent excessively large dot product values. The normalized weights are obtained by applying Softmax(·) to the scaled scores, which are subsequently multiplied by the value matrix *V* to produce the output features. The multi-head attention mechanism employs *h* attention heads capable of parallel computation. Each attention head ${\mathrm{head}}_{i}$ independently performs the aforementioned attention computation. The outputs of all attention heads are concatenated and then linearly projected to generate the final output sequence. $W^{Q}$, $W^{K}$, and $W^{V}$ denote the weight matrices for queries, keys, and values, respectively, while $W^{O}$ represents the linear projection weight matrix for the output. Subsequent DWCViT modules adopt the same architecture but introduce downsampling to reduce the spatial dimensions of feature maps while expanding the channel dimensions, thereby achieving hierarchical feature representation.

**Figure 6:**
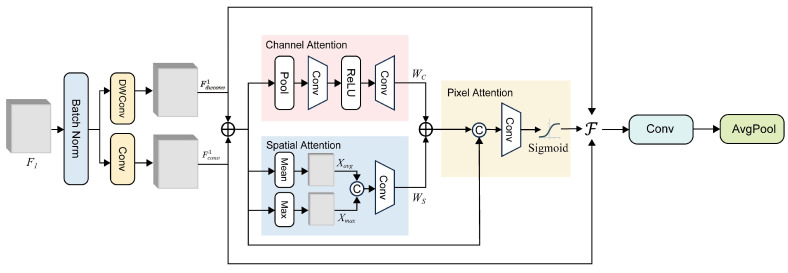
Diagram of the DWCA module. DWConv denotes depth-wise separable convolution, and the ⨁ symbol represents element-wise addition.

Two identical DWCA modules are cascaded after DWCViT, with their structure illustrated in Figure 6. The feature map $F_{1}\in R^{B\times H_{1}\times W_{1}\times C_{1}}$ output by DWCViT serves as the input to the first DWCA module. Similar to the DWCViT module, batch normalization is first applied to the input features. Subsequently, the features are processed through a depth-wise separable convolution and a standard convolution, yielding two feature maps $F_{dwconv}^{1}{\in R}^{B\times H_{1}\times W_{1}\times C_{2}}$ and $F_{conv}^{1}{\in R}^{B\times H_{1}\times W_{1}\times C_{2}}$. These two feature maps are fused through element-wise addition to obtain the combined feature map $F_{f}^{1}$, enabling the capture of richer feature representations. The fused feature map is fed into dual-attention mechanism computation branches to calculate the channel attention weights $\;W_{C}$ and spatial attention weights $W_{S}$.

(14) $$\begin{array}{c} W_{C}=W_{2}*ReLU\left(W_{1}*GAP\left(F_{f}^{1}\right)+b_{1}\right)+b_{2} \end{array}$$

(15) $$\begin{array}{c} W_{S}=W_{3}*Concat\left(X_{avg},X_{max}\right)+b_{3} \end{array}$$

GAP(·) denotes the global average pooling, $W_{1}$ represents the convolutional weights, $b_{1}$ is the bias term, and similarly for $W_{2}$ and $b_{2}$. $X_{avg}\in R^{B\times H_{1}\times W_{1}\times C_{2}}$ represents the result of taking the mean along the channel dimension, while $X_{max}\in R^{B\times H_{1}\times W_{1}\times C_{2}}$ denotes the result of taking the maximum value along the channel dimension. Concat(·) denotes channel-wise concatenation. $W_{3}$ and $b_{3}$ represent the convolutional weights and biases, respectively. The fused attention weights, which incorporate both spatial positional information and channel-wise information, are obtained by summing the weight matrices. This hybrid weight matrix is then concatenated with the previously obtained fused feature map $F_{f}^{1}$ after dimensionality expansion. This process generates a new feature representation that preserves foundational feature information while enhancing critical features. The weight $W_{p}$ is derived through convolutional operations and a Sigmoid activation function. The entire pixel attention module generates these more refined feature weights that further improve the feature extraction ability. Finally, the resultant features are obtained via the F function, followed by convolution and average pooling operations.

(16) $$\begin{array}{c} F\left(F_{dwconv}^{1},F_{conv}^{1},W_{p}\right)=F_{f}^{1}+W_{p}*F_{dwconv}^{1}+\left(1-W_{p}\right)*F_{conv}^{1} \end{array}$$

In Equation (16), the term $\left(1-W_{p}\right)$ adaptively modulates the contribution ratio between $F_{dwconv}^{1}$ and $F_{conv}^{1}$.

#### 3.3.3. MAFM

**Figure 7:**
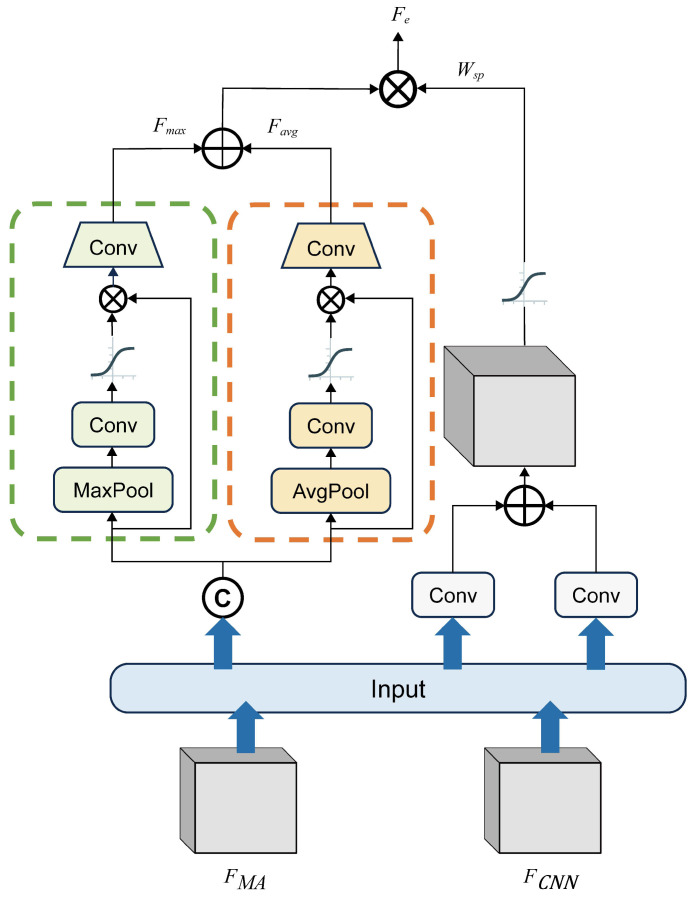
Schematic diagram of the MAFM structure. The symbol ⨂ denotes element-wise multiplication and ⨁ denotes element-wise addition. The activation function used here is the Sigmoid function. The pathways in the two dashed boxes are used to extract channel features.

The Multi-path Adaptive Feature Module serves as a feature fusion module following the feature extraction stage. It adaptively integrates distinct critical features from the input feature maps that have the same scale through three parallel pathways. It generates enhanced feature maps to facilitate improved feature learning and processing in subsequent modules, thereby boosting the network’s detection accuracy. The detailed architecture of this module is illustrated in Figure 7.

The MAFM receives feature maps from the MA-Branch and CNN Branch, denoted as $F_{MA}$ and $F_{CNN}$, with dimensions $F_{MA}\in R^{B\times H_{MA}\times W_{MA}\times C_{MA}}$ and $F_{CNN}\in R^{B\times H_{CNN}\times W_{CNN}\times C_{CNN}}$, respectively, where $H_{MA}=H_{CNN}$ and $W_{MA}=W_{CNN}$. In the first pathway, apply a 1×1×1 convolution followed by a Sigmoid activation function to each input feature map, generating spatial attention weights $W_{sp}$ to highlight salient regions. The convolutions project the channel dimension to 1, indicating we only focus on the spatial dimensions. After using traditional additive fusion, the activation function is used to extract the important position weights in space.

(17) $$\begin{array}{c} W_{sp}=Sigmoid\left(Conv\left(F_{MA}\right)+Conv\left(F_{CNN}\right)\right) \end{array}$$

As for the second and third pathways, the two input feature maps are directly concatenated along the channel dimension to produce the combined feature map $F_{concat}\in R^{B\times H_{c}\times W_{c}\times \left(C_{MA}+C_{CNN}\right)}$, where $H_{C}=H_{CNN}$ and $C_{MA}=C_{CNN}$. To enable subsequent networks to utilize the concatenated feature map while emphasizing critical features along the channel dimension, we designed two parallel channel-aware pathways for deep feature fusion. These pathways share identical structures except for the type of pooling layer employed. They both get the spatial weights first and then get the weighted feature map. The global max pooling in the first pathway extracts the most discriminative features representing object categories to reduce classification loss, while the global average pooling preserves the average information of the feature map. Subsequently, channel-wise attention weights are extracted through convolutional layers and a Sigmoid activation function, which are then element-wise multiplied with the concatenated feature map $F_{concat}$ to produce weighted feature maps. To reduce channel dimensionality for compatibility with downstream modules, dimensionality-reduction convolutional layers are introduced, generating the output feature maps $F_{avg}$ and $F_{max}$ from the two pathways. Finally, the feature maps from the parallel pathways are fused through element-wise addition and multiplied by the spatial attention weight $W_{sp}$, yielding enhanced features that integrate both local information and contextual information. The entire process described above can be represented by the following mathematical formula:

(18) $$\begin{array}{c} F_{avg}^{\prime }=Sigmoid\left(Conv\left(AVGPool(F_{concat})\right)\right)*F_{concat} \end{array}$$

(19) $$\begin{array}{c} F_{avg}=Conv_{1}\left(F_{avg}^{\prime }\right) \end{array}$$

(20) $$\begin{array}{c} F_{max}^{\prime }=Sigmoid\left(Conv(MAXPool\left(F_{concat}\right))\right)*F_{concat} \end{array}$$

(21) $$\begin{array}{c} F_{\max\;}={\mathrm{Conv}}_{1}\left(F_{\max\;}^{\prime }\right) \end{array}$$

(22) $$\begin{array}{c} F_{e}=W_{sp}*F_{max}+F_{avg} \end{array}$$

$AVGPool\left(\cdot \right)$ denotes average pooling, and $MaxPool\left(\cdot \right)$ represents max pooling. The convolution layers used after the pooling layers have the shape of 1×1. ${Conv}_{1}\left(\cdot \right)$ refers to the convolutional layers at the end of the two parallel pathways, used for channel-wise dimensionality reduction.The input channel dimension is twice as large as the output. The final enhanced feature $F_{e}\in R^{B\times H_{e}\times W_{e}\times C_{e}}$ retains the same spatial dimensions and channels as the input feature maps. The entire MAFM module functions as a plug-and-play structure. In this study, since the original CNN architecture employs a Feature Pyramid Network (FPN) that generates multi-scale feature maps, while subsequent modules like the RPN and Region of Interest (RoI) Pooling operate on feature maps of varying resolutions, we strategically apply MA-Branch and CNN Branch feature fusion only to the 20×20×256 feature maps to balance computational cost and detection accuracy. For feature maps of other scales, internal feature fusion within the CNN branch is performed.

### 3.4. Experimental Settings

#### 3.4.1. Evaluation Metric

To comprehensively evaluate the performance of the proposed model in this paper, we employ common evaluation metrics for few-shot object detection, including precision, recall, F1 score, and mean Average Precision (mAP). Precision reflects the reliability of the model’s predictions and can be expressed by the following formula:

(23) $$\begin{array}{c} \;\mathrm{Precision}\;=\frac{TP}{TP+FP} \end{array}$$

True Positive (*TP*) represents the number of correctly detected positive samples, i.e., the count of targets accurately identified by the model. False Positive (*FP*) denotes the number of non-target objects mistakenly classified as targets by the model. The precision value ranges within $\left[0,\;1\right]$, where a value closer to 1 indicates fewer false positives and higher prediction reliability. However, the limitation of precision lies in its failure to account for instances where the model misses actual targets. To address this, the recall metric is introduced to measure how well the model detects all relevant targets. Its formula is

(24) $$\begin{array}{c} \mathrm{Recall}=\frac{TP}{TP+FN} \end{array}$$

False Negative (*FN*) stands for the number of samples that the model incorrectly predicted as non-targets. The value of recall also ranges from 0 to 1. The higher the value, the more comprehensive the model is in identifying target objects. Although precision and recall have different emphases, for object detection tasks, the combination of the two can comprehensively measure the model’s balance between accurate identification and comprehensive coverage. F1 score is a balance between precision and recall, and it can comprehensively evaluate the performance of the model:

(25) $$\begin{array}{c} F_{score}=\frac{\left(B^{2}+1\right)\cdot \mathrm{Precision}\cdot \mathrm{Recall}}{B^{2}\cdot \mathrm{Precision}+\mathrm{Recall}} \end{array}$$

$F_{score}$ is the harmonic mean of precision and recall. When B=1, it yields the F1 score. For the F1 score, if either precision or recall is significantly low, it imposes a substantial penalty, resulting in a low F1 score. Additionally, Average Precision (AP) is a widely used evaluation metric in object detection tasks. It represents the area under the precision-recall curve, mathematically defined as follows:

(26) $$\begin{array}{c} AP=\int_{0}^{1}P\left(R\right)\;dR \end{array}$$

$P\left(R\right)$ represents the precision and recall curve. The value range of AP is also within $\left[0,\;1\right]$. For multi-class object detection tasks, AP values are generally calculated for each category first and then averaged to obtain the final AP. In this study, AP50 is selected as the evaluation index. The formula is as follows:

(27) $$\begin{array}{c} IoU=\frac{DR\cap GT}{DR\cup GT} \end{array}$$

(28) $$\begin{array}{c} AP50=\frac{1}{N}\sum_{i=1}^{N}AP_{i}^{@50} \end{array}$$

DR represents the predicted bounding box, and GT represents the ground truth. For each category to be detected, the average precision at IoU=0.5 is first calculated, and then the average value of AP of all categories is obtained, which is AP50. AP50 is a relatively lenient evaluation index, but it can quickly evaluate the performance of the model, so it is widely used. Finally, for the few-shot strategy of this paper, we also evaluated the AP50 of all classes and the nAP50 of novel classes. AP50 can reflect the model’s detection ability for all leaf diseases. nAP50 fully reflects the generalization ability of the model on few-shot data. Through the above evaluation indicators, the performance of the model is quantitatively analyzed from multiple dimensions, and a comprehensive performance evaluation system is formed, which provides a basis for the analysis of subsequent experimental results.

#### 3.4.2. Hardware and Software Platform

The experiments were conducted on a server equipped with an NVIDIA GeForce RTX 2080 Ti GPU featuring 22 GB of VRAM, suitable for graphics computing and deep learning tasks. The CPU is an Intel Xeon Platinum 8336C. It has a high number of threads and can achieve high-speed storage collaboration with the GPU. The system is configured with 45 GB of RAM and a storage setup comprising a 30 GB SSD system drive and a 50 GB data drive, capable of handling rapid I/O operations for large-scale datasets. The software platform is built on the Detectron2 framework, a PyTorch-based deep learning framework specialized in object detection and instance segmentation tasks. This supports mixed precision training, reduces video memory usage, and improves throughput. It integrated an ONNX export function that facilitates model deployment to production environments. Here, we chose the version of 0.3 that leverages CUDA 11.0 and cuDNN 8.0 for accelerated training. The Python version is 3.8, which is widely used by many deep learning frameworks. The PyTorch framework’s version is 1.7.0, which is compatible with our hardware setting and is very stable. The operating system is Ubuntu 18.04 LTS, a Linux-based platform providing long-term support, good stability, and compatibility with mature drivers. Numpy 1.21.2 is used for high-dimensional operations. We use TensorBoard to monitor the training process in real time and visualize it for easy and intuitive observation. Detailed environment specifications are provided in Table 4.

#### 3.4.3. Optimizer and Hyperparameter Settings

In the experiments, the leaf disease detection dataset was partitioned into training, validation, and test sets in an 8:1:1 ratio. Following the progressive data partitioning method proposed by Bingyi Kang et al. [48], the dataset was further divided into base-class and full-class subsets based on data usage during the pre-training and fine-tuning phases. For the fine-tuning phase, K-shot random sampling (K = 1, 3, 5, 10) was performed per class. All the images are resized to 640×640 pixels. The proposed model employs the stochastic gradient descent (SGD) optimizer due to its excellent performance in deep learning tasks. The optimizer with a momentum of 0.9 and a weight decay of 0.0001, according to experimental experiences. During the initial training stage, the batch size is set to 10, while in the fine-tuning stage, it is reduced to 4. The max iteration is set to 5000 in the pre-training stage and 25,000 in fine-tuning. A learning rate scheduling strategy combining warm-up and multi-step decay is adopted. The base learning rate is 0.02 for pre-training and 0.0004 for fine-tuning. The warm-up strategy gradually increases the learning rate from zero to the base learning rate over the first few epochs, avoiding unstable gradient updates at the early stage. A uniform warm-up of 100 iterations is applied across phases to mitigate gradient explosion risks during early training. After many iterations, the multi-step decay is deployed. It reduces the learning rate by a fixed factor (we set the γ rate at 0.1) at predefined milestones (we set 4000 at the pre-training stage and 30,000 at the fine-tuning stage), enabling coarse-to-fine optimization. A larger batch size and base learning rate will make the pre-training stage more stable and foster convergence. All hyperparameter configurations are empirically determined through extensive experimentation and ensure the model’s superior performance in leaf disease datasets.

#### 3.4.4. Comparison Methods

To evaluate the performance of the method proposed in this paper in few-shot tea disease detection, we select some classic and novel works in the field of few-shot object detection as the models for comparative experiments. These models include FsDet [36], CD-ViTO [49], DeFRCN [50], Meta Faster R-CNN [31], Deformable DETR [51], DETReg [41]. Each model adopts different solutions and has outstanding performance in different scenarios. FsDet is a classic few-shot object detection network based on pre-training methods, which surpasses many meta-learning methods in detection effect. CD-ViTO is a newly proposed transformer-based cross-domain few-shot object detection network, which proposes learnable instance features, domain prompters, and other structures to significantly improve the robustness of cross-domain detection. DeFRCN introduces a gradient decoupling layer and prototype calibration block into the original Faster RCNN network, which further alleviates the potential contradictions of the original model in few-shot object detection tasks. The Meta Faster R-CNN network introduces prototype matching into RPN and the classifier from the perspective of object proposals so as to generate proposals of new categories efficiently and effectively. As an innovative transformer-based detector, Deformable DETR demonstrates two key advancements: first, its hierarchical multi-scale design significantly improves small object detection accuracy; second, the integration of deformable convolutional operations effectively addresses the slow convergence issue inherent in standard transformer architectures. DETReg is a self-supervised approach that inherits from DETR. It pre-trains the whole object detection model to match the localizations and align the feature embeddings. And then fine-tunes downstream tasks. By comparing the method proposed in this paper with the above methods, the effect of model detection of tea diseases can be fully reflected. All comparative experiments were carried out on the same dataset and its partitioned datasets. The detection indexes included recall, precision, nAP50, and F1 score mentioned in Section 3.4.1. Since all the above models are multi-stage training models, the base learning rate, number of iterations, batch size, and other parameters of all models in the K-shot fine-tuning stage are kept the same to ensure the fairness of the comparative experiment.

## 4. Results and Discussion

### 4.1. Disease Detection Results

The objective of this experiment is to compare the capabilities of different few-shot object detection models for tea leaf disease detection. Under 5-shot and 10-shot scenarios, we evaluate and analyze each model’s ability to mitigate data sparsity and extract features in complex environments using metrics, including nAP50, recall, and precision.

**Figure 8:**
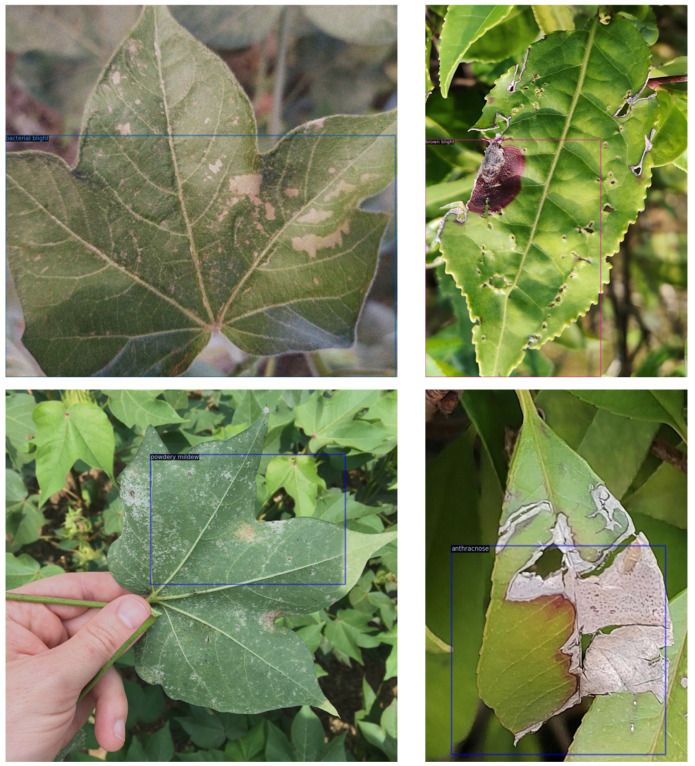
The detection results of MAF-MixNet. The left column is the cotton leaves, and the right column is the tea leaves.

As shown in Table 5, our proposed model comprehensively outperforms other compared few-shot detection models in precision, nAP50, and F1 score, achieving optimal overall performance. Across these three metrics, our model surpasses baseline models and the novel CD-ViTO model by nearly 20%. This demonstrates that our model, through multi-level feature extraction and enhancement, effectively addresses challenges posed by limited samples and complex backgrounds, enabling accurate localization and classification of tea leaf diseases. The top three rows of the table, based on Faster R-CNN variants, exhibit higher false positive rates, potentially due to limitations in purely convolutional feature extraction networks. Meta Faster R-CNN achieves suboptimal nAP50 primarily because its meta-learning approach alleviates calibration and classification biases. The CD-ViTO and Deformable DETR models are both transformer-based models. The CD-ViTO model shows a competitive F1 score and precision by leveraging Vision Transformer to capture long-range dependencies, improving classification accuracy. However, its overall performance remains constrained by sample scarcity and background complexity. Deformable DETR also shows excellent results on F1 score and precision, but the score of nAP50 is much higher than CD-ViTO. We assume that maybe the multi-scale feature maps extracted by the backbone of Deformable DETR are vitally important for detection accuracy. Our model extracts the multi-scale using the CNN branch and leverages the transformer-based branch at the same time. Further improved the model’s capabilities in the feature extraction stage. DETReg and Deformable DETR use the same detector but different backbones. Deformable DETR shows better performance on all the metrics. We assume that the backbone is vitally important for few-shot object detection models. Because the effect of feature extraction gained from backbones plays a decisive role in the detection results. Similarly, in Table 6, our model exceeds all others in precision, nAP50, and F1 score. Notably, MAF-MixNet achieves 73.8% nAP50, significantly outperforming baseline models. These results robustly validate our model’s broad applicability in agricultural disease detection. The detection results of our model are demonstrated in Figure 8. We can see that although our model classifies different categories correctly, its localization remains limited, with bounding boxes deviating from actual object edges.

Joint analysis of Table 5 and Table 6 reveals that all ResNet-based models exhibit varying degrees of degradation in precision, nAP50, or F1 score. This may stem from inherent limitations of the pure CNN-based architecture or noise amplification with increased data volume. Nevertheless, our proposed model effectively suppresses such degradation, demonstrating strong noise robustness and superior detection capabilities. This proves our two-branch architecture is effective to a certain extent. Furthemore, we find out that although the DETReg method only uses a shallow feature extraction backbone structure, it can surpass the FsDet network in the 10-shot scenario. This shows that the self-supervised training method can effectively improve the performance of the model in scenarios with more data. The models in the last four rows of the tables all use the transformer structure, but our proposed model performs better, which shows that the global and local feature extraction methods we proposed achieve mutual complementation of features, and MAFM achieves effective feature fusion enhancement.

### 4.2. Ablation Experiments

**Figure 9:**
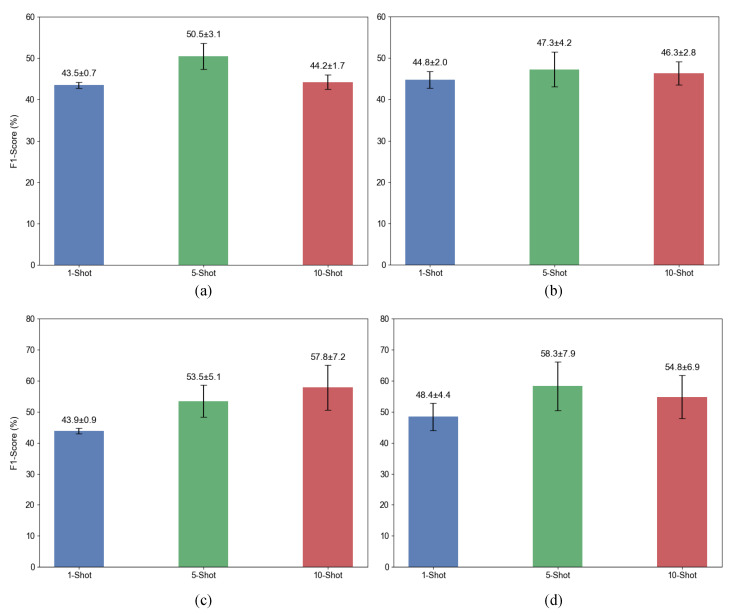
Mean F1 score and standard deviation for the four ablation experiment groups. (**a**) corresponds to the baseline model, (**b**) is the baseline model with MA-Branch added, (**c**) is the baseline model with MAFM added, and (**d**) is our proposed model.

To validate the effectiveness of the proposed MA-Branch and MAFM components, we conducted four ablation experiments: directly using the FsDet baseline model, adding MA-Branch to the baseline model, adding MAFM structure to the baseline model, and using MA-Branch and MAFM structure on the baseline model at the same time. It should be noted that in the experiment of introducing MA-Branch to the basic model, in order to make the subsequent module able to obtain the features extracted by this branch, a simple addition fusion method that does not change the size of the feature map is adopted to ensure that the data in the network can be transmitted correctly. All experiments use ResNet-101 as the backbone network, and the evaluation indexes are nAP50 and F1 score. The results of the ablation experiment are shown in Table 7 and Figure 9.

From the Table 7, it can be seen that the addition of MA-Branch to the baseline model has improved performance in K-shot (K=1,3,5,10) scenarios, with an increase of 16% in nAP50 in the 10-shot scenario. This indicates that the MA-Branch can enhance the richness and overall quality of features extracted by the model, guiding the model to locate diseased areas. The addition of the MAFM module to the original network increased the nAP50 to 27.2%, 40.3%, 57.0%, and 69.6%, respectively, demonstrating that the module can effectively enhance feature expression and improve the overall performance of the model. Finally, by adding both MA-Branch and MAFM to the original model, the results in the last row of the table show that the nAP50 value far exceeds the other three groups of experiments. This indicates that the modules we designed are not only effective individually but also work better together, achieving satisfactory detection results.

### 4.3. Validation of Model Robustness

To evaluate the generalization ability and robustness of the model we proposed, we designed this verification experiment. In this experiment, we used part of the data in the IP102 [52] crop pest dataset. This is because some of the label files in the original dataset link are lost due to time constraints. We found that the number of samples of 102 kinds of pests in the original data set is not balanced. Therefore, we selected images of *aphids* (*Aphis gossypii* Glover), *xylotrechus* (*Anoplophora glabripennis* (Motschulsky)), *flea beetles* (*Phyllotreta striolata* (Fabricius)), and *legume blister beetles* (*Epicauta chinensis* Laporte) on crops such as *rice* (*Oryza sativa* L.), *corn* (*Zea mays* L.), and *wheat* (*Triticum aestivum* L.) as the data set for the verification experiment. Among them, the number of images of pests is 879, 1265, 37, and 50, respectively. Based on *aphids* and *tiger beetles* as the base classes, the other two are the new classes. To ensure the fairness of the experiment, the partitioning process of the corresponding few-shot data set of this pest data set is the same as that of the tea disease data set. Crop pests are very different from tea diseases in shape, size, color, and texture. The appearance of different growth stages of insects also has certain differences. Compared with the original dataset, the pest dataset also has a larger sample size, and the samples are more diverse and complex. The images in this dataset also have complex backgrounds, but the crop background is quite different from the primary *tea* background, so the model needs to have better adaptability to complex backgrounds. Therefore, if it can also perform well on this dataset, this can prove that the model does have strong stability. The robustness verification results are shown in Table 8. The evaluation metrics used include recall, precision, AP50, and F1 scores. The MAF-MixNet we proposed achieves 51.6% recall, 26.9% precision, 56.3% AP50, and 34.1% F1 score, which are better than other models on AP50 and F1 score. These results indicate that MAF-MixNet has a certain robustness and can be applied to other agricultural datasets. This is attributed to the model’s ability to extract and enhance both detailed features and global features. In contrast, the precision of the FsDet and DeFRCN models is not satisfactory. From the perspective of model structure analysis, it may be because neither of them has designed a critical information extraction module, making the detection susceptible to background noise interference. Moreover, the training steps of DeFRCN are complex, and the decoupled structure requires a large number of training parameters. The Meta Faster RCNN model shows good results in AP50 and recall, which is attributed to the design of the Meta RPN structure of the network, which greatly reduces the number of missed detections. The inherent trade-off between recall and precision often leads to misjudgments in the model. Deformable DETR and DETReg show better results in precision compared with the Faster RCNN-based architecture. We believe that the DETR detector used by both is more effective.

### 4.4. Computational Efficiency Analysis

In actual agricultural implementations, deploying models to edge computing devices for real-time inference frequently encounters computational resource limitations. Traditional deep learning models often fail to meet real-time demands due to their high computational costs and inference latency. Under such constraints, an effective and practical model should achieve an optimal balance between detection accuracy and computational efficiency. Therefore, this chapter explores the feasibility of agricultural edge deployment by quantitatively analyzing the computational efficiency. Table 9 evaluates the computational scalability of the models from three perspectives: floating-point operations (FLOPs), parameter count, and inference speed. FLOPs measure the computational complexity by quantifying the number of floating-point operations required for one forward propagation. To comprehensively assess memory usage across differently trained models, the parameter count reported here represents the total parameters, including both trainable and frozen parameters. The inference speed indicates the processing time per image with identical resolution (640 × 640) on the same hardware platform (NVIDIA 2080 Ti).

As evidenced in the table, our model demonstrates computational complexity comparable to CD-ViTO and Deformable DETR, both including transformer architecture, while exhibiting higher FLOPs than purely convolutional models. Notably, the Meta Faster R-CNN model with a purely convolutional backbone shows exceptionally high computational complexity. This can be attributed to its meta-learning framework, which employs a dual-branch architecture for simultaneous support and query feature extraction, along with a non-linear RPN and attention-based feature alignment classifier, collectively increasing architectural complexity. DETReg achieves the second largest FLOPs, which is partly due to the computational complexity brought by the transformer structure itself and partly due to the introduction of the self-supervised training method. Regarding parameter count and inference speed, our model shows a substantial parameter size while maintaining mid- to-high-range inference speed. Further analysis reveals that the MA-Branch structure accounts for the majority of parameters (15.16M parameters remain after its removal). This parameter accumulation likely stems from employing two Vision Transformer structures alongside a ResNet-101 backbone, combining their parameter counts. The relatively moderate impact on inference speed and computational complexity may be explained by the separation of the two Vision Transformers, thus contributing twice the number of non-compute-intensive parameters. Comparative analysis indicates that Deformable DETR achieves the optimal accuracy-efficiency trade-off among all evaluated models. While Fsdet shows superior computational metrics (FLOPs and parameters), its detection performance proves inadequate. Our model delivers outstanding detection accuracy but suffers from excessive parameter size, directing our future optimization efforts toward parameter reduction. This guides us to adopt strategies to further optimize the number of model parameters. We can try methods such as quantization and model compression, similar to the work of Zainab Ameema and Syed Dabeeruddin [53], to reduce the number of model parameters and achieve real-time detection of the model on low-computing power devices like the Raspberry Pi. Alternatively, we can use a lightweight backbone structure similar to MobileViT [54].

### 4.5. Limitation and Future Work

Although the proposed model has demonstrated its superiority in the task of tea disease detection in multiple experiments, there are still some defects that are worth further research and improvement. Firstly, from the perspective of training strategy, although the proposed method realizes detection on K-shot novel class data, it still relies on a large amount of supervised base class data training in the early stage. In fact, it is very time-consuming and laborious to produce a high-quality annotated dataset. Therefore, self-supervised or semi-supervised learning methods can be tried to reduce the dependence on labeled data. Although we have analyzed and performed some experiments on recent research, we failed to change our pretraining pattern to the self-supervised and semi-supervised pattern. Secondly, the data enhancement method used in this paper is relatively basic and cannot generate more diversified data. The pretraining dataset is still very small compared with public datasets such as ImageNet and MS-COCO, which are used to train large models. Moreover, some fuzzy and deformed data are deleted in the process of data processing, which makes the model’s adaptability to dynamic scenes weaker and reduces the upper limit of the model’s generalization performance to some extent. To this end, the data set can be further expanded by fusing cross-category features or context-aware generation methods. Lastly, the model’s parameter count is extremely large compared with the baseline. This makes our model not enough to meet the deployment requirements of low-power edge devices. We can try some quantification methods or change the backbone as mentioned in Section 4.4.

Our forthcoming research efforts will focus on the following aspects: We first plan to further explore model deployment optimization and adaptation to actual application scenarios. In the future, we plan to alter the proposed method’s backbone to a more lightweight backbone. Since there are many well-known lightweight networks, such as YOLO [55], SSD [56], and MobileNet [57], models based on these networks have been widely used in the deployment of edge computing devices. We try to use or learn and modify their structures to obtain a more efficient model. Deploying the trained model to the edge device for execution. Furthermore, we tend to employ various sparse attention mechanisms to substantially reduce model parameters while maintaining detection accuracy. Second, we try to implement class-incremental learning approaches to enhance real-world applicability, enabling continuous model adaptation to expanded detection categories while mitigating catastrophic forgetting on base classes. Finally, the research method in this paper is limited to a single 2D visual task. In order to achieve a wider range of applications, the input of the model can be extended to video or 3D data processing, and the recognition can be extended from a closed world to an open world. With the upsurge of the large multi-modal models, the visual tasks can be combined with text prompts to use the strong generalization ability of text semantics to guide the positioning of new categories.

## 5. Conclusions

In traditional agricultural production, extensive management is mainly adopted, and the detection of diseases and pests depends heavily on manual labor. Due to the lack of professional agricultural knowledge, the judgment of diseases and pests is inaccurate, and the treatment is not scientific. Not only has the quality of agricultural products declined and production decreased, but farmers have also caused great agricultural product safety problems by indiscriminate use of pesticides. With the proposal of the concepts of precision agriculture and smart agriculture, how to detect plant diseases and insect pests with high quality and efficiency has become an issue of concern in the industry. Computer vision technology, with its efficient feature extraction capabilities and non-destructive detection characteristics, has become a feasible solution for agricultural disease detection.

This paper proposes a few-shot end-to-end tea disease detection network, MAF-MixNet, which has both local and global feature extraction capabilities, as well as key feature enhancement functions. By designing a mixed attention mechanism branch, MA-Branch, which is parallel to the traditional convolution branch, the contextual information is efficiently extracted. Then, in order to couple and optimize the extracted features, we use the multi-path feature fusion module MAFM with adaptive capabilities as a bridge between the double branch, as well as the backbone and the follow-up module. A large number of experiments have proved that MAF-MixNet has efficient detection capability and generalization ability. In the comparative experiments with the other six models, our model achieved the best precision, nAP50, and F1 score results in both 5-shot and 10-shot scenarios, with nAP50 reaching 60.1% and 73.8%, respectively. In the ablation experiment, the two modules we proposed have improved over the original model in any-shot scenarios. Finally, considering the needs of a wider range of applications, we evaluate the proposed model’s robustness and scalability. We use a public crop pest dataset for training and testing on different models and achieve an AP50 of 56.3% in the 5-shot scenario, far exceeding other models. In order to evaluate the feasibility of the model in real agricultural scenarios, we calculated the computational complexity, number of parameters, and inference speed of our model. The experiment shows that our model is acceptable in terms of inference speed and computational complexity, but the number of parameters is pretty large. This provides a new direction for our future research.

In summary, the contributions of this paper are reflected in the following points: First, we have created a leaf disease detection dataset dedicated to few-shot learning. The dataset supports progressive dataset partitioning and expands the existing few-shot dataset based on the training paradigm of base classes and novel classes. Secondly, a new mixed attention mechanism feature extraction branch and feature fusion module are proposed so that the entire network shows good accuracy and stability in the disease detection task. Finally, through a large number of experimental analyses of the advantages and disadvantages of some classic and novel models in handling few-shot complex background tasks, it provides a reference for follow-up research.

## Figures and Tables

**Table 1:** Distribution of the original dataset.

Category	Number
Tea anthracnose disease	25
Tea brown blight disease	28
Cotton fusarium wilt disease	185
Cotton powdery mildew	258

**Table 2:** Data cleaning.

Category	Number (Before Data Cleaning)	Number of Blurred Images	Number of Redundant Images	Number (After Data Cleaning)
Fusarium wilt disease	185	63	37	68
Powdery mildew	258	182	0	64
Anthracnose disease	25	4	2	19
Brown blight disease	28	4	3	21

**Table 3:** Data Augmentation.

Category	Number (Before Augmentation)	Number (After Augmentation)
Fusarium wilt disease	68	408
Powdery mildew	45	270

**Table 4:** Experimental Environment Configuration.

Components	Version
Detectron2	0.3
PyTorch	1.7.0
Python	3.8
Numpy	1.21.2
CUDA	11.0
TensorBoard	2.6.0

**Table 5:** Comparative Experiments under 5-shot Setting. We mark the best results in bold.

Model	Backbone	Recall	Precision	nAP50	F1 score
FsDet	ResNet101	**84.0**	34.2	40.4	48.6
Meta Faster RCNN	ResNet101	73.9	35.9	53.4	48.3
DeFRCN	ResNet101	81.0	33.0	47.9	46.9
CD-ViTO	ViT-L	83.1	40.4	40.3	54.3
Deformable DETR	ResNeXt-101+DCN	83.9	41.5	44.7	55.5
DETReg	ResNet50	79.2	28.0	32.7	41.4
MAF-MixNet (ours)	ResNet101+ViT-L	70.2	**62.0**	**60.1**	**65.9**

**Table 6:** Comparative Experiments under 10-shot Setting. We mark the best results in bold.

Model	Backbone	Recall	Precision	nAP50	F1 score
FsDet	ResNet101	80.3	32.6	14.3	46.4
Meta Faster RCNN	ResNet101	**84.9**	43.8	52.6	57.8
DeFRCN	ResNet101	49.3	37.3	33.4	42.5
CD-ViTO	ViT-L	81.9	43.5	43.5	56.8
Deformable DETR	ResNeXt-101+DCN	83.9	48.6	54.1	61.7
DETReg	ResNet50	83.4	34.2	50.9	48.5
MAF-MixNet (ours)	ResNet101+ViT-L	63.5	**63.6**	**73.8**	**63.6**

**Table 7:** Ablation Study of All Components. We mark the best results in bold.

Model	MA-Branch	MAFM	1-Shot nAP50	3-Shot nAP50	5-Shot nAP50	10-Shot nAP50
Baseline	✗	✗	14.0	21.1	40.4	14.3
	✓	✗	23.5	26.4	41.4	30.3
	✗	✓	27.2	40.3	57.0	69.6
Ours	✓	✓	**34.1**	**53.5**	**60.1**	**73.8**

**Table 8:** Experimental Results of 5-shot Pest Detection. We mark the best results in bold.

Model	Recall	Precision	AP50	F1 score
FsDet	78.9	19.9	45.9	31.8
Meta Faster RCNN	**90.3**	18.6	53.8	30.8
DeFRCN	75.6	15.0	31.9	25.1
Deformable DETR	62.0	23.3	23.3	33.9
DETReg	53.8	23.8	32.4	33.0
MAF-MixNet (ours)	51.6	**26.9**	**56.3**	**34.1**

**Table 9:** Comparison of computational efficiency. We bold the results with the largest values.

Model	FLOPs(G)	Params	Inference Speed (FPS)
FsDet	34.51	14.50	7.75
Meta Faster RCNN	**169.00**	39.81	5.24
DeFRCN	83.90	51.93	2.44
Deformable DETR	86.05	32.54	**18.46**
CD-ViTO	94.90	51.93	5.88
DETReg	158.84	39.83	4.29
MAF-MixNet (ours)	87.22	**241.40**	11.63

## Data Availability

The leaf disease detection dataset supporting the findings of this study is openly available in Hugging Face Datasets. This dataset contains 718 annotated images of tea and cotton leaves across four disease categories (Tea Anthracnose Disease, Tea Brown Blight Disease, Cotton Fusarium Wilt Disease, and Cotton Powdery Mildew), formatted as JPEG with accompanying XML metadata. The resource can be accessed via the DOI: https://doi.org/10.57967/hf/5174 or directly at https://huggingface.co/datasets/ttkqwe123/leaf_disease_detection (accessed on 4 March 2025).
